# Field Implementation of Precision Livestock Farming: Selected Proceedings from the 2nd U.S. Precision Livestock Farming Conference

**DOI:** 10.3390/ani14071128

**Published:** 2024-04-07

**Authors:** Yang Zhao, Brett C. Ramirez, Janice M. Siegford, Hao Gan, Lingjuan Wang-Li, Daniel Berckmans, Robert T. Burns

**Affiliations:** 1 Department of Animal Science, The University of Tennessee, Knoxville, TN 37996, USA; 2 Department of Agricultural and Biosystems Engineering, Iowa State University, Ames, IA 50011, USA; 3 Department of Animal Science, Michigan State University, East Lansing, MI 48824, USA; siegford@msu.edu; 4 Department of Biosystems Engineering and Soil Science, The University of Tennessee, Knoxville, TN 37996, USA; 5 Department of Biological and Agricultural Engineering, North Carolina State University, Raleigh, NC 27695, USA

Precision Livestock Farming (PLF) involves the real-time monitoring of images, sounds, and other biological, physiological, and environmental parameters to assess and improve animal health and welfare within intensive and extensive production systems. Precision Livestock Farming systems have the potential to empower farmers to make better management decisions, based upon objective measures, during an animal production cycle. The potential benefits from PLF systems can only be realized if these systems are adopted in practice by livestock and poultry producers. The advancement of research in digital agriculture, and, more specifically, PLF technologies, over the past decade has been very impressive; however, the field implementation of PLF technology has not occurred at the same rate as research advancements.

The United States land grant university system is well equipped to interface between PLF research and on-farm adoption by disseminating research to farmers through the Extension Service component of the system. The on-farm implementation of PLF systems in the United States has the potential to provide increased animal production efficiency with improved animal health and welfare and human working conditions, while also increasing the economic, environmental, and social sustainability of animal production systems. Significant improvements in the production efficiency of all food animal sectors will be required in order to meet the anticipated growth in demand for animal proteins by a vaster worldwide population over the next 30 years. The commercial adoption of PLF systems is integral to achieving the increases in production efficiency needed from our livestock sectors and to providing needed data to animal production stakeholders. 

For this reason, the theme of the 2nd U.S. Precision Livestock Farming conference (USPLF2023) was “Field Implementation of PLF”. This emphasizes the significance of implementing technology in food animal production to meet the rising demand for animal products in the future. The USPLF2023 conference was held from 21 to 24 May 2023 at The University of Tennessee Conference Center in Knoxville, TN. The conference was organized by The University of Tennessee, University of Nebraska-Lincoln, and Tennessee State University.

The goal of the USPLF2023 conference was to help better equip researchers, Extension practitioners, technology providers, and livestock and poultry producers to adopt PLF systems on working farms across the United States and abroad. The USPLF2023 conference provided a timely and essential platform for industry professionals and academia to share knowledge, network, and collaborate on all aspects of animal management using innovative technology. The conference also made scholarships available for participation from farmers, minority-serving institutions educators, researchers, and Extension specialists, thus providing opportunities to a diverse group of attendees to benefit from the conference’s valuable insights and discussions. In addition, the event hosted two other important meetings, namely the United States Department of Agriculture (USDA) National Institute of Food and Agriculture (NIFA) Inter-Disciplinary Engagement in Animal Systems Project Director meeting, and the meeting of the NC1211 Multi-state project (Precision Management of Animals for Improved Care, Health, and Welfare of Livestock and Poultry).

The USPLF2023 conference featured four primary research topics: Sensors and Sensing in PLF [1,2,3,4,5], Data Management and Algorithm Development [6,7,8], Measuring, Modeling, and Managing of Dynamic Responses [9,10,11,12,13,14], and Societal Impacts of PLF [15,16]. A total of 126 submissions were received for these topics from individuals representing universities, research institutions, and PLF companies from 13 different countries across Africa, Asia, Australia, Europe, North America, and South America. The submissions underwent two rounds of rigorous review by ad hoc reviewers to assess their relevance, originality, readability, and formatting. Out of these submissions, 114 were accepted for presentations at the conference, either orally or as poster presentations. 

From the accepted submissions, the conference proceedings committee carefully selected the highest-ranked papers to be included in this Special Issue with the full endorsement of the Conference Proceeding Committee. Additionally, several voluntarily submitted proceedings were also considered and incorporated into this Special Issue. Each paper within this collection represents a significant contribution, showcasing the advancements made and the future potential of implementing PLF in the field.

This Special Issue is expected to be of immense value to individuals working in the interdisciplinary fields of animal science, agricultural engineering, computer science, electrical engineering, and veterinary science. It represents valuable resources for educators, researchers, Extension specialists, animal producers, and industry leaders who share a commitment to advancing the livestock and poultry industry in an innovative and sustainable manner.
